# PROTOCOL: Police initiated diversion for youth to prevent future delinquent behavior: a systematic review protocol

**DOI:** 10.1002/CL2.208

**Published:** 2018-05-25

**Authors:** David B. Wilson, Iain Brennan, Ajima Olaghere

## Background

### The problem, condition or issue

Misbehavior is a normal part of adolescence and that misbehavior sometimes crosses the line from disruptive or problematic to delinquent. Nationally representative surveys of youth in the United States have indicated that minor delinquent behavior is normative, particularly for boys (Elliott et al., 1983). The normative nature of minor delinquent behavior raises the question of how police should respond to minor delinquent behavior in a way that is corrective, but also avoids involving the youth in the criminal justice system beyond what will be effective in reducing future misbehavior. Stated differently, what is the right level of response to minor delinquent acts? Overly punitive responses may have the unintended consequence of increasing the likelihood of future delinquency; overly lenient responses may fail to serve as a corrective for the misbehavior. Police diversion schemes are a collection of strategies police can apply as an alternative to formal processing of youth. Police initiated diversion schemes aim to reduce reoffending by steering youth away from deeper penetration into the criminal justice system and by providing an alternative intervention that can help youth address psychosocial or other needs that contribute to their problem behavior.

Diversion as an option is popular among law enforcement officers, as it provides an option between ignoring youth engaged in minor wrongdoing and formally arresting such youth. Diversion has the potential to reduce reoffending by limiting the exposure of low‐risk youth to potentially harmful deviant peers within the criminal justice system. Furthermore, diversion may reduce criminal justice system costs, freeing these resources for higher risk youth. However, some commentators (Ray & Childs, 2015; Mears et al., 2016) have noted diversion may widen the population of youth under the surveillance of the criminal justice system if youth are subsequently punished for failing to meet the terms of their diversion. Consequently, diversion may inadvertently increase youth reoffending. The uncertain potential for diversion to produce both benefits and harms and law enforcement's sustained use of diversion underscores the importance of comprehensively reviewing the effectiveness of these interventions.

### The intervention

Police‐led juvenile diversion is a pre‐court intervention initiated by police that represents an alternative to formal processing or the imposition of formal charges. Stated differently, this review will focus on the pre‐charge diversion of youth. Examples might involve a caution, a restorative caution, or a final warning or reprimand. Each of these alternatives might be combined with an additional program element such as referral to a treatment service provider. Police‐led diversions may be known by many names, such as cautions, final warnings, police‐led intervention, police control of juveniles, police‐led proactive prevention, police‐led diversion, pre‐charge diversion or simply as diversion. The essential feature is the intervention is initiated and led by police and the youthful offender receives a diversionary scheme to avoid a criminal record and any negative consequences that may result from continued formal contact with the criminal justice system (e.g, imposition of formal charges, conviction, etc.).

The essential feature of a traditional police cautioning scheme involves a police officer, the youth in question, and the parents, at a minimum. Victims are not involved nor do police officers receive any training, but solely provide an explanation about the legal and social consequences of continued delinquent behavior. However, variants of this scheme can involve other interventions and services (Audit Commission, 1996) or involvement of a script of certain questions to structure discussion between an offender and the affected parties and the presence of the victim, in the case of restorative cautioning or conferencing (Wilcox et al., 2004). As for the final warning and reprimand scheme, this involves an assessment‐based approach to evaluate the seriousness of the offense and, depending on the gravity of the offense, a reprimand or final warning with referral to a multi‐agency team for further assessment and placement in a behavioral treatment program (Holdaway, 2003, p. 352).

### How the intervention might work

Wilson and Hoge (2015) articulate two theoretical supports for diversion: labeling theory and differential association theory. Labeling theory posits that the stigmatizing effect of labeling a youth as delinquent may establish expectations for future delinquent acts and alter that youth's social networks toward more deviant peers, thus increasing the likelihood of future deviant behavior (Bernburg et al., 2006). Thus, diverting a low‐risk youth not already labeled as “delinquent” may reduce future offending. Sutherland's (1939) differential association theory states a youth learns the values, attitudes, and techniques of criminal behavior through the interaction with delinquent peers. As such, diverting low‐risk youth from the juvenile justice system may reduce exposure to deviant peers. In essence, a youth who has engaged in a delinquent act may learn more serious forms of delinquency from others already in the juvenile justice system, including the values and attitudes that support involvement in delinquency.

Another theoretical mechanism underlying diversion is the reintegrative shaming aspect of restorative justice. Reintegrative shaming emphasizes an intervention scheme where responses to transgressions are de‐stigmatizing and inclusive of “a meaningful community‐based process that reaffirms the boundaries of acceptable behavior” (Zhang, 2011, p. 2325). These responses should aim to reduce or inhibit new or further stigmatization as a result of contact with the justice system. Forgiveness and non‐stigmatization are central principles of reintegrative shaming, as these tenets reinforce another core feature—reintegration. Reintegration concerns efforts to restore offenders (and victims alike) after a transgression and reintegrate them into the community. This process also implicates communities of care such as significant others, e.g., family members or individuals of import in an offender's life who are central to disavowing unlawful behavior and facilitating forgiveness. Our interpretation of community of care includes that of authority figures such as police officers, whom we understand may not traditionally be viewed as members of an affected person's broader prosocial community. Yet, the interaction between the police officer and the youth provides an opportunity for an authority figure to reinforce appropriate norms by briefly detaining the youth engaged in a problem behavior, thus enacting an element of shame. The shame may be enhanced by the police officer taking the youth to her home and discussing the youth's acts with her parent. Additionally, the element of reintegration emerges with diverting the youth from any further formal processing, allowing the youth to return to a state of “good standing” within the community (Hay, 2001; Sherman, 1993).

Referral to needed and effective services is the final mechanism that might contribute to the effectiveness of some diversion schemes. As discussed above, some diversion programs involve formal referrals to treatment services or a needs assessment for services. As the first point of contact with the justice system, police officers are poised to intervene early and provide referrals to needed services that may be more beneficial in reducing future delinquent acts than a formal criminal justice sanction (Butts, 2016; Mears et al., 2016).

Diversionary practices, however, may also be harmful in the sense of increasing a youth's propensity to engage in delinquent behaviors. From a deterrence perspective, diversion provides a swift sanction, but the sanction may be too mild to deter a youth from similar (or more severe) behavior in the future. A youth who perceives that he was not held responsible for his actions may think that he “got away with it” and will continue to engage in similar ways.

Mears et al. (2016) also suggest a possible “net widening” effect of diversion programs. When a diversion with conditions is used instead of a diversion with ‘no further action’ and a youth fails to meet the specified conditions, such as attending an appointment with a counselor, the youth may be brought into the criminal justice system as a consequence. This may result in a low‐risk youth experiencing negative consequences of juvenile justice system involvement.

### Prior reviews

Several meta‐analyses of diversion programs exist and these differ from each other and from the proposed review in important ways. In a Campbell Collaboration review, Petrosino et al. (2010) examined the effectiveness of juvenile justice system processing compared to any alternative non‐system condition. Petrosino et al. (2010) found that formal processing produced worse outcomes than diversion from the system. Their focus, however, examined whether justice system processes were beneficial and as such did not differentiate pre‐ versus post‐charge diversion nor examine other features of the diversion programs. Similarly, Wilson and Hoge (2015) examined 45 studies and found that on average the diversion conditions had lower recidivism rates than formal judicial processing. Additionally, their analysis showed slightly larger beneficial effects for pre‐charge diversion compared to post‐charge diversion. However, methodologically stronger research designs failed to find a positive effect for diversion relative to traditional processing. Finally, a meta‐analysis completed by Schwalbe et al. (2012) focused on diversion programs with a treatment component such as case management, family treatment, youth court, etc. And although not explicitly stated, these diversion programs were likely post‐charge. The findings showed a small overall effect favoring these programs, but the effects were not statistically significant except for family treatment. Overall, Schwalbe et al. (2012) do not provide a meaningful examination of police‐led diversions, which are the focus of the current review.

Taken as a whole, these meta‐analyses provide an equivocal answer regarding the effectiveness of diversion programs. Furthermore, these prior reviews did not specifically focus on pre‐charge or police‐led diversion. Based on labeling and differential association theories, we would expect diversion at this stage to be more effective as it avoids any labeling of the youth, even if temporarily via a formal charge and at a minimum, reduces potential exposure to deviant peers in the juvenile justice system. Hence, the purest form of diversion occurs at this stage per the avoidance of any juvenile justice system processing.

Despite the uncertainty regarding the effectiveness of diversionary practices, police officers widely use diversion schemes. Precise estimates of the prevalence of diversion are difficult given that the central feature of diversion involving no formal charge reduces documentation by the justice system. Consequently, prevalence estimates of police‐led youth diversions are rare or must be extrapolated to national levels from small area studies. Additionally, definitions of diversion vary by jurisdiction, further impeding accurate estimates of prevalence. According to a Ministry of Justice study, 21% of youth arrests in England and Wales results in a caution (Ministry of Justice, 2016). Within this system, these cautions still form part of a youth's juvenile record, so it is debatable whether these constitute diversion as discussed above. In the United States, Puzzanchera and Kang (2008) estimate that a similar number (25%) of youth entering the juvenile justice system are diverted, but much of this is initiated post‐charge, rather than by the police officer during the initial interaction with the youth.

Departing from, but building on the work of prior reviews, we will focus our systematic review and meta‐analysis on police‐led diversion prior to the imposition of formal charges. This narrower focus will help inform police practice and use of diversion. Furthermore, we will explore the differential effectiveness of the various diversionary schemes, such as a diversion with no further action, restorative caution, or diversion with various therapeutic elements.

## Objectives

The objective of this review is to synthesize the evidence on the effectiveness of pre‐court interventions involving police warning, reprimand, and cautioning schemes in reducing delinquent behavior. Our specific research questions are:

1. Are police‐initiated diversions effective in reducing future delinquent behavior (i.e., additional cautioning, arrest, court appearances, or findings of guilt)?
2. Is effectiveness related to the type of police‐initiated diversion used (i.e., traditional cautioning, caution plus, police restorative cautioning, final warning or reprimand)?
3. Is effectiveness related to characteristics of the youth (i.e., age, gender, race/ethnicity, crime committed, and offense history)?

## Methodology

### Criteria for including and excluding studies

#### Types of study designs

Both experimental and quasi‐experimental designs will be included. The specific eligibility for each design is detailed below.

Experimental designs. Eligible experimental designs must have randomly assigned participants to a diversion or a control condition(s). Designs that used a quasi‐random assignment procedure, e.g., assignment based on an alternate case basis, are also eligible.

Quasi‐experimental designs. Several types of quasi‐experimental designs are eligible; however, all quasi‐experimental designs must have a comparison group that is similar to the police diversion intervention group with respect to demographic characteristics and prior involvement in delinquent behavior (i.e., be at similar risk for future delinquent behavior). This similarity can be achieved through matching or statistical controls. Matching may be at the individual level or at the group level. Statistical control methods include regression analysis, analysis‐of‐covariance, and propensity score modelling, among others. Use of a statistical control method is sufficient for inclusion meaning, we will not exclude studies based on a subjective assessment of the quality of the statistical controls. Rather, any quasi‐experimental design that controls for baseline risk factors, such as age, gender, and prior offense history, will be eligible. Quasi‐experimental designs are not eligible if the comparison group is comprised of participants who refused participation in a police diversion scheme or who dropped out of a police diversion scheme. Quasi‐experimental designs that do not have a comparison group are not eligible.

#### Types of participants

The population of interest are youth suspected of involvement in a crime or delinquent behavior. Eligible studies must have included participants who were youth between 12 and 17 years of age, inclusively. Participant samples that included a small proportion (i.e., less than 20%) of youth over 17 but less than 22 are also eligible. Participants must also have been apprehended, arrested, or otherwise referred to the juvenile justice system, and either diverted to a police‐involved intervention prior to the imposition of formal charges, or in the case of a comparison condition, treated in some other fashion.

#### Types of interventions

Interventions that will be considered eligible must have been initiated and implemented by police officers as identified by the study. This includes programs where diversion occurs any time prior to formal charges—whether before or after arrest—but prior to the imposition of formal charges. Interventions that involve court or prosecutorial referrals, even with the inclusion of police officers, will not be considered eligible. Findings will be considered relevant if measured at the police ‘level of referral’. Control conditions will typically be ‘treatment as usual’, which is often a process of laying criminal charges followed by adjudication through the criminal justice system. Studies where a disposal of ‘no further action’ has been treated as a control condition will be excluded.

#### Types of outcome measures

The primary outcome of interest is delinquency. Eligible studies must report at least one delinquency‐related outcome. This may include official measures of delinquency, such as an arrest, or other measures of delinquent‐type behaviors, such as self‐report, parent‐report, or school records of wrongdoing. Secondary outcomes of interest include self‐report measures related to improved relations, such as satisfaction with police or the cautioning process.

### Search strategy

Four categories of key words were developed for this search. The first category lists key terms and synonyms related to youth and their social status. The second category of key terms are related to pre‐court cautioning practices and schemes. The third and fourth categories address the methodology and the measured study outcomes, respectively. Zotero, a reference management software program will be used to retrieve, store, and document the search process. Each database will have its own file folder within Zotero and will be searched individually. Search notes will be created for each database and stored in the appropriate file folder. The search notes will capture: the date of the search, the database name, the final search string used, the reference yield produced, and a notes field to capture any aberrant issues.

1. Population
  youth OR child OR juvenile OR delinquent OR devian* OR student OR adolescent OR “young person” OR “young offender*” OR bully* OR “youthful offender”
2. Treatment
  diverted OR diversion OR caution* OR “caution plus” OR restorative OR “restorative caution” OR triage OR “final warning” OR reprimand OR “alternative* to custody” OR “pre‐charge” OR “pre‐caution” OR “pre‐court” OR “pre‐custody” OR “alternative program*” OR disposal OR disposition OR liaison OR Police‐led OR “police initiated” OR “police control” OR “police diversion” OR police OR “law enforcement” OR “civil citation”
3. Methodology
  outcome OR evaluat* OR effect OR effectiv* OR experiment* OR quasi OR assessment OR RCT OR “random* control*”
4. Outcome
  recidivism OR arrest* OR rearrest* OR citation OR offend* OR reoffend* OR conviction OR reconviction OR adjudication OR adjudicated

### Electronic sources

The search strategy described above will be applied to the following databases, which cover both the easily accessible sources as well as the grey literature.

Australian Institute of Criminology

Center for Problem Oriented Policing

CINCH (the Australian Criminology Database) via Informit

Criminal Justice Abstracts

EconLit

First Search—Dissertation Abstracts

Global Policing Database

Google Scholar

HeinOnline

Home Office (including archives)

Ministry of Justice

NCJRS (National Criminal Justice Reference Service)

Peter Neyroud's Database (list of RCTs)

Policy Archive

PolicyFile

Criminal Justice Periodicals (now ProQuest Criminal Justice)

Dissertations & Theses: Full Text

OVID

PubMed

PsycINFO

ProQuest Dissertations and Theses

Public Affairs Information Service

RAND Documents

Safetylit.org

Social Sciences Citation Index

Social Services Abstracts

Sociological Abstracts

SSRN—Social Science Research Network

Worldwide Political Science Abstracts

In addition to searching the electronic resources listed above, we will also scan the references of relevant reviews and identified studies, and consult with an information search specialist and experts in the field.

### Criteria for determination of independent findings

The primary unit‐of‐analysis for this review will be a research study defined as a distinct sample of study participants involved in a common research project. Multiple reports (e.g., publications, technical reports, etc.) from a common research study will be coded as a single study. Stated differently, a research study will only be treated as unique if the study sample does not include study participants included in any other coded study. Multiple effect sizes will be coded, if possible, from each studies. Statistical independence will be maintained or modeled in all statistical analyses. The primary analysis of the effect of diversion on delinquency will use the most general measure of delinquency reported in each study that is closest to a one‐year post‐diversion time point. Secondary analyses will explore whether any observed diversion effects increase or decrease over time, whether the effects differ across different measures of delinquency, etc. These analyses will handle the issue of statistical dependencies by using the method of robust standard errors developed by Hedges, Tipton, and Johnson (2010).

### Details of study coding categories

The following categories of information will be coded for eligible studies: study characteristics, intervention/comparison characteristics, outcome characteristics, and effect size data. Coding will be unique for each eligible study, as the unit of analysis for the meta‐analysis is an independent study. In cases where there are multiple publications for the same study, the most complete study will be coded as the primary study and all other related publications will be coded as cross‐references. Methodological quality and risk of bias will be coded as data is extracted for study, intervention/comparison, and outcome characteristics. Specifically, at the study level, we will code for the type of experimental and quasi‐experimental design based on assignment (e.g., matching, wait list control, cohort, etc.). Risk of bias will be captured by assessing the risk of selective outcome reporting. At the intervention/comparison level, risk of bias will be coded based on reported or observed differences between groups at baseline (selection bias) and attrition bias for the primary outcome, in terms of quantity and differential attrition. Finally, at the outcome level risk of bias will be based on one item, which will capture whether there is potential bias from non‐blinding procedures.

### Statistical procedures and conventions

The primary outcome for this review is delinquency and is most often reported on a dichotomous scale, that is, as delinquent or non‐delinquent. As such, the effect size of choice for this review will be the odds ratio. Odds ratios will be computed from any available information such as proportions, percentages, raw frequencies, chi‐square and marginal distributions, etc. In the case of quasi‐experimental designs with statistical adjustments for baseline differences, the regression coefficient from a logistic regression model will be coded as the logged odds ratio along with the reported standard error. Effect sizes based on scaled measures of delinquency will be computed as *d*‐type effect sizes and then converted to odds ratios using the logit transformation method (Lipsey and Wilson, 2001). All effect size computations will be established equations as implemented in the online effect size calculator available on the Campbell website.

Meta‐analysis will be conducted using random effects models estimated via full‐information maximum likelihood. Primary analyses will be performed using Stata packages developed by David B. Wilson and available at http://mason.gmu.edu/~dwilsonb/ma.html. The robust standard error method of modeling statistical dependences will be implemented with the Stata package *robumeta* (see http://www.northwestern.edu/ipr/qcenter/RVE‐meta‐analysis.html for details). Moderator analyses of a single categorical variable will be fit using the analog‐to‐the‐ANOVA method, also under a random effects model. Moderator analyses of continuous moderators or of multiple moderators will be conducted with meta‐analytic regression methods, also under a random effects model.

Publication‐selection bias will be assessed in three ways. First, analyses will compare the results from published and unpublished reports. Published documents will include peer‐reviewed journal articles, books, and book chapters. All other report forms, such as theses, technical reports, government and agency reports, will be considered unpublished. Second, we will perform a trim‐and‐fill analysis on the primary delinquency outcome. Third, we will visually inspect a funnel plot on the primary delinquency outcome.

### Planned moderator analyses

Our *a priori* planned moderator analyses include the type of diversion (e.g., traditional cautioning, caution plus, police restorative cautioning, final warning or reprimand), the type of research design (e.g., experiment versus quasi‐experiment), country of intervention, and publication type (i.e., published versus unpublished). *Post hoc* moderator analyses will explore the relationship between other study features and effect size.

### Treatment of qualitative research

We do not plan to include qualitative research.

## Review authors

**Lead review author:**

The lead author is the person who develops and co‐ordinates the review team, discusses and assigns roles for individual members of the review team, liaises with the editorial base and takes responsibility for the on‐going updates of the review.

**Table:** 

**Name:**	**David B. Wilson**
Title:	Professor and Department Chair
Affiliation:	George Mason University
Address:	354 Enterprise Hall, MS 4F4
City, State, Province or County:	Fairfax, Virginia
Postal Code:	22030
Country:	USA
Phone:	+001 (703) 993‐4701
Email:	dwilsonb@gmu.edu
**Co‐authors:**	
**Name:**	**Iain Brennan**
Title:	Senior Lecturer
Affiliation:	University of Hull
Address:	Cottingham Road, Room 225a, Wilberforce Building
City, State, Province or County:	Hull
Postal Code:	HU6 7RX
Country:	UK
Phone:	+44 (0) 1482 465717
Email:	I.Brennan@hull.ac.uk
**Name:**	**Ajima Olaghere**
Title:	Postdoctoral Research Fellow
Affiliation:	George Mason University
Address:	354 Enterprise Hall, MS 4F4
City, State, Province or County:	Fairfax, Virginia
Postal Code:	22030
Country:	USA
Email:	aolagher@gmu.edu

## Roles and responsibilities

- Content: David Wilson has extensive background knowledge on juvenile justice programs. Iain Brennan has experience with executing a research grant on a restorative justice program and has recently completed an evaluation of a police‐led diversion scheme. Ajima Olaghere, along with David Wilson, are currently working on a meta‐analysis focusing on restorative justice programs for youth.
- Systematic review methods: David Wilson has extensive expertise in systematic review methods. Ajima Olaghere has worked on meta‐analyses with David Wilson and previously worked with Catherine Gallagher on systematic reviews. Iain Brennan has led a systematic review of interventions to reduce violence in licensed premises.
- Statistical analysis: David Wilson has developed tools that are in wide use for performing the statistical analyses related to meta‐analysis. He also authored a book on these methods with Mark Lipsey.
- Information retrieval: David Wilson, Ian Brennan and Ajima Olaghere all have experience performing systematic searches on various topics and retrieving studies and documents for review.

## Sources of support

This systematic review is supported by funding from the Jacobs Foundation.

## Declarations of interest

We have no potential conflicts of interest with respect to this review.

## Preliminary timeframe

**Table:** 

May 16, 2016	Submit title registration form to Campbell
June 1	Submit protocol to Campbell
July 1	Start literature search
July—August	Literature search
July—August	Revise protocol in response to Campbell review
September	Abstract screening studies for eligibility
September	Develop coding database
October—January	Coding, double coding, resolving coding differences
February— March	Analyze data, generate tables and figures
April—May	Draft report
June 1	Submit draft review to Campbell and the Jacobs Foundation
July—August 2017	Revise as needed based on Campbell and Jacobs review

## Plans for updating the review

This review will be updated every four years and updating it will be the primary responsibility of David Wilson unless all authors agrees that another author take primary responsibility.

## Author declaration

### Authors' responsibilities

By completing this form, you accept responsibility for preparing, maintaining and updating the review in accordance with Campbell Collaboration policy. The Campbell Collaboration will provide as much support as possible to assist with the preparation of the review.

A draft review must be submitted to the relevant Coordinating Group within two years of protocol publication. If drafts are not submitted before the agreed deadlines, or if we are unable to contact you for an extended period, the relevant Coordinating Group has the right to de‐register the title or transfer the title to alternative authors. The Coordinating Group also has the right to de‐register or transfer the title if it does not meet the standards of the Coordinating Group and/or the Campbell Collaboration.

You accept responsibility for maintaining the review in light of new evidence, comments and criticisms, and other developments, and updating the review at least once every five years, or, if requested, transferring responsibility for maintaining the review to others as agreed with the Coordinating Group.

### Publication in the Campbell Library

The support of the Coordinating Group in preparing your review is conditional upon your agreement to publish the protocol, finished review, and subsequent updates in the Campbell Library. The Campbell Collaboration places no restrictions on publication of the findings of a Campbell systematic review in a more abbreviated form as a journal article either before or after the publication of the monograph version in *Campbell Systematic Reviews*. Some journals, however, have restrictions that preclude publication of findings that have been, or will be, reported elsewhere and authors considering publication in such a journal should be aware of possible conflict with publication of the monograph version in *Campbell Systematic Reviews*. Publication in a journal after publication or in press status in *Campbell Systematic Reviews* should acknowledge the Campbell version and include a citation to it. Note that systematic reviews published in *Campbell Systematic Reviews* and co‐registered with the Cochrane Collaboration may have additional requirements or restrictions for co‐publication. Review authors accept responsibility for meeting any co‐publication requirements.

**I understand the commitment required to undertake a Campbell review, and agree to publish in the Campbell Library. Signed on behalf of the authors**:

## Study Level Coding Form

This coding form is for each unique study. Note that a study may be reported in multiple manuscripts (publications, technical reports, etc.). Also, some reports may include the results for distinct studies, such as evaluations in different cities. Our unit‐of‐analysis for the meta‐analysis is an independent study. No two studies should include any of the same participants. If there are multiple publications for the same study, use the most complete study as the primary study ID and all other related studies as cross reference IDs.

## Comparison Level Coding Form

This coding form is for each treatment/comparison contrast coded from a study. For most studies, you will only code this form once. However, some studies may have two or more treatment conditions or two or more comparison conditions. In the coding below, it is critical to indicate if any of the treatment/comparison contrasts for a study share sample participants. For example, a study might have two distinct treatments but only one comparison group. In this case, these comparisons share sample participants (i.e., the same comparison condition).

## Outcome (Dependent Variable) Coding Form

Code each eligible outcome or dependent variable using the form below. Note that you should code this only once for a variable that is measured at multiple time points. That is, recidivism measured at 3, 6, and 9‐months is a single dependent variable. Code the characteristics of the measure using this form and the data for each measurement time point on the effect size forms.

## Effect Size Coding Form

Code all effect sizes of interest using the form below, coding each effect size separately (i.e., with a different copy of the form or record in the database). Indicate the study ID, the comparison ID, and the dependent variable ID. Give each effect size within a study a unique idea, numbering sequentially (1, 2, 3 …).

There are several ways to compute effect sizes using the different tabs. ONLY USE ONE METHOD per effect size. If you have the raw means and also a regression coefficient for the same outcome from a model that adjusts for baseline differences, these are two different effect sizes. The different effect size computation methods are:

1. Means and standard deviations
2. Means and standard errors
3. Frequency of failures in each condition
4. Proportion of failures in each condition
5. Logistic regression coefficient for treatment effect dummy code
6. OLS unstandardized regression coefficient
7. OLS standardized regression coefficient
8. Independent samples t‐test
9. Chi‐square test (2 by 2, df = 1)
10. Point‐biserial correlation coefficient
11. Phi correlation coefficient
12. Hand computation (e.g., using the online effect size calculator)
